# LncRNA Gm35585 transcriptionally activates the peroxidase EHHADH against diet-induced fatty liver

**DOI:** 10.1038/s12276-025-01420-5

**Published:** 2025-03-13

**Authors:** Ming Jin, Qian Lu, Ninglin Xia, Xue Fan, Ziling Zhang, Xiaofei Huang, Li Sun, Luyong Zhang, Zhenzhou Jiang, Qinwei Yu

**Affiliations:** 1 New Drug Screening and Pharmacodynamics Evaluation Center, Jiangsu Center for Pharmacodynamics Research and Evaluation, State Key Laboratory of Natural Medicines, China Pharmaceutical University, Nanjing, China; 2 Center for Drug Research and Development, Guangdong Pharmaceutical University, Guangzhou, China; 3 Key Laboratory of Drug Quality Control and Pharmacovigilance, Ministry of Education, China Pharmaceutical University, Nanjing, China

**Keywords:** Mechanisms of disease, Non-alcoholic fatty liver disease, Biological therapy

## Abstract

Metabolic-dysfunction-associated steatotic liver disease is one of the most common chronic liver diseases worldwide and has no approved treatment thus far. Here we report that the hepatic overexpression of Gm35585, a novel lncRNA downregulated in the livers of mice fed a high-fat diet, is functionally important in alleviating hepatic lipid accumulation pathologies. Gm35585 activates the peroxisome proliferator-activated receptor α (PPARα) signaling pathway and promotes the expression of downstream PPARα-target gene, enoyl-CoA hydratase and 3-hydroxyacyl CoA dehydrogenase (EHHADH), which is one of the four enzymes of the peroxisomal β-oxidation pathway. Activation of EHHADH promotes the oxidation of long-chain fatty acids (LCFAs), and the increased levels of hepatic LCFAs contribute to metabolic-dysfunction-associated steatotic liver disease. Mechanistically, Gm35585 binds to retinoid X receptor α (RXRα) and then forms a PPARα/RXRα heterodimer with PPARα and guides the heterodimer to recognize the promoter of EHHADH, which is called peroxisome proliferator-activated receptor response element, causing transcriptional activation of EHHADH. Taken together, Gm35585 is a hepatic lipid metabolism regulator that activates EHHADH transcription, promoting peroxisomal β-oxidation of LCFAs and ultimately ameliorating diet-induced fatty liver.

## Gm35585 regulates lipid metabolism in fatty liver disease

Metabolic-dysfunction-associated steatotic liver disease (MASLD) is a growing global health issue affecting millions and leads to severe liver conditions. Researchers explored new ways to treat MASLD by studying a molecule called Gm35585. In their study, they used mice fed a high-fat diet to mimic human liver disease. They found that Gm35585 helps to break down harmful fats in the liver by activating a protein called EHHADH, which is involved in fat metabolism. This process reduces fat accumulation in the liver, potentially slowing disease progression. They concluded that Gm35585 could be a promising target for developing new MASLD treatments. Their findings suggest that enhancing the activity of EHHADH through Gm35585 might offer a new therapeutic strategy. Future research could focus on how this approach can be applied to human treatments for fatty liver disease.

This summary was initially drafted using artificial intelligence, then revised and fact-checked by the author.

## Introduction

The prevalence of metabolic-dysfunction-associated steatotic liver disease (MASLD) has continued to rise globally in the past two decades and has replaced viral hepatitis as the number one chronic liver disease worldwide^1^. Without pharmacological intervention, MASLD can progress to metabolic-dysfunction-associated steatohepatitis and eventually to fibrosis, cirrhosis and even hepatocellular carcinoma^2,3^. Studies have shown that 240 million people are expected to suffer from MASLD by 2025, and the risk to human health and the socioeconomic burden are evident^4^. Due to the heterogeneity of its pathogenesis, clinical manifestations and pathological changes, there is no uniform treatment plan for MASLD, and the main treatment measures are weight loss, diet control and exercise^5^. Furthermore, no drugs targeting the treatment of MASLD have been marketed. Although pharmacotherapies are in development, the response rates are modest^6^. Therefore, investigating the regulatory mechanisms of MASLD may provide new drug targets for its treatment, which is of great clinical significance.

Although obesity and insulin resistance contribute to lipolysis, which is an important cause of MASLD, increased long-chain fatty acid (LCFA) uptake is a direct cause of lipid accumulation in hepatocytes^7^. Studies have shown that, under high-fat diet (HFD) conditions, the body ingests a large amount of lipid components, which causes increased uptake of LCFAs in hepatocytes, one of the important causes of HFD-induced fatty liver^8^. The accumulation of LCFAs in hepatocytes has been reported to induce apoptosis, an inflammatory response and oxidative stress and to induce MASLD^9–11^. However, medium-chain fatty acids (MCFAs) do not cause such pathological development^12^. Therefore, accelerating hepatic LCFA catabolism to form MCFAs and preventing the intracellular accumulation of LCFAs are key to slowing the development of MASLD induced by a HFD.

The peroxisome is an integral part of fatty acid (FA) β-oxidation in LCFAs, and activation of peroxisomal FA β-oxidation could be a potential therapeutic strategy for HFD-induced MASLD^13^. The second hydration and third dehydrogenation steps in the peroxisomal β-oxidation system are catalyzed by a bifunctional protein of two types in mammalian cells, D-bifunctional protein and L-bifunctional protein (also known as enoyl-CoA hydratase and 3-hydroxyacyl CoA dehydrogenase, EHHADH), respectively^14,15^. Among them, D-bifunctional protein is present in the liver, brain and other tissues, and EHHADH is mainly present in the liver^16^. Studies have shown that hepatic EHHADH expression is decreased in mice with HFD-induced fatty liver^17^, suggesting that EHHADH may be a key regulator of MASLD.

In recent years, the role played by long noncoding RNAs (lncRNAs) in metabolic diseases has gradually gained increasing attention^18^. For instance, NEAT1, lnc-uc372 and MEG3 are reportedly involved in lipid metabolism processes^19^. These reports highlight that lncRNAs may be utilized as nucleic-acid-based therapeutics for metabolism-related diseases. Nevertheless, most of these RNAs are competitive endogenous RNAs that can bind competitively to microRNAs, leading to gene silencing. The biological roles of potential lncRNAs through other regulatory approaches have yet to be determined.

Here, we identified a novel lncRNA, Gm35585, as a positive regulator of hepatic LCFA degradation. This study investigated the regulatory role of Gm35585 in hepatic lipid accumulation induced by a HFD, proposes a novel regulatory mechanism of lipid metabolism and provides a new theoretical basis and potential therapeutic target for MASLD treatment.

## Materials and methods

### Animal treatment

The animal experiments in our study conformed with the animal care laws and guidelines. All procedures were approved by the Animal Care and Use Committee of China Pharmaceutical University (no. 2022-05-019). The experimental animals were housed at room temperature, maintained at 20–25 °C and 50–70% humidity, and provided free access to food and water.

Ten diet-induced obesity (DIO) mice and five C57BL/6J mice were purchased from Huachuang Sino Pharmaceutical Technology for the Gm35585 and EHHADH overexpression experiments, respectively. The adeno-associated viruses AAV-Gm35585 (4.37 × 10^12^ vg/ml), AAV-NC (negative control, a adeno-associated virus empety vector) (1.8 × 10^11^ vg/ml), TBG-EHHADH (1.02 × 10^12^ vg/ml) and TBG-NC (5.78 × 10^13^ vg/ml) were manufactured by GenePharma. All mice were male, and the DIO mice were 5-week-old C57BL/6J mice subjected to 10 weeks of high-fat feeding with 60% fat (Medicience) to establish a HFD model before Gm35585-related adeno-associated virus transfection. By contrast, 5-week-old C57BL/6J mice were subjected to 4 weeks of high-fat feeding with 60% fat before EHHADH-related adeno-associated virus transfection.

The following three groups were used for each experiment: for Gm35585 overexpression: (1) the normal control diet (NCD) + AAV-NC group, in which five C57BL/6J mice were fed a NCD for 15 weeks and administered AAV-NC; (2) the HFD + AAV-NC group, in which five DIO mice were fed a HFD and administered AAV-NC; and (3) the HFD + AAV-Gm35585 group, in which five DIO mice were fed a HFD and administered AAV-Gm35585. For EHHADH overexpression, (1) in the NCD + TBG-NC group, five C57BL/6J mice were fed an NCD for 9 weeks and administered TBG-NC; (2) in the HFD + TBG-NC group, five DIO mice were fed a HFD and administered TBG-NC; and (3) in the HFD + TBG-EHHADH group, five DIO mice were fed a HFD and administered TBG-EHHADH. Finally, the animals were euthanized by orbital bleeding under anesthesia. Tissues were rapidly collected, immediately frozen on dry ice and then transferred to −80 °C.

### Cell treatment

In vitro experiments were performed using the AML-12 cell line (Procell) and HepG2 cell line, which were cultured with complete AML-12 medium (Procell) and complete Dulbecco’s modified Eagle medium (containing 10% FBS, 1% streptomycin–penicillin and 1 mM sodium pyruvate) (Gibco), respectively. The pEX-3-Gm35585 overexpression plasmid and RNA oligonucleotides (small interfering RNAs (siRNAs)) used for cell experiments were obtained from GenePharma. The oligonucleotide sequences are presented in Table 1.

**Table 1:** The oligonucleotide sequences for siRNA experiments in vitro.

siRNA	Sense (5′ to 3′)	Antisense (5′ to 3′)
NC	UUCUCCGAACGUGUCAGGUTT	ACGUGACACGUUCGGAGAATT
Gm35585	GGGCAGAUAUUUGCUUUAUTT	AUAAAGCAAAUAUCUGCCCTT
Ehhadh	GUUGCUUAUAUUCCCUUAUTT	AUAAGGGAAUAUAAGCAACTT
Rxrα	GAGCCAUUGUCCUGUUCAATT	UUGAACAGGACAAUGGCUCTT

An in vitro hyperlipidemic model was established using 0.2 mM palmitic acid (PA) (Sangon Biotech) administered for 24 h. For plasmid overexpression experiments, Lipofectamine 3000 (Thermo Fisher) was used to transfect the pEX-3-Gm35585 plasmid (0.4 μg per 5,000 cells) for 24 h, and the same dose of pEX-3 empty vector was used as a negative control. For RNA interference experiments, RNA oligonucleotides (0.8 μM per 2 × 10^4^ cells) were transfected with Lipofectamine 3000 for 24 h. For siRNA and pEX-3-Gm35585 coadministration, RNA interference was performed, followed by overexpression of Gm35585. GW7647 and GW6471 (MedChemExpress) are PPARα-specific agonists and inhibitors, respectively. GW7647 (5 μM) was used to agonize PPARα by treatment for 24 h, followed by treatment with 0.2 mM PA. After coadministration of the siRNAs, cellular RNA interference was performed before GW7647 was administered. The pEX-3-Gm35585 plasmid and 0.2 mM PA were added after the cells were treated with 1 μM GW 6471 for 24 h.

### RNA sequencing

Total RNA from tissue samples was extracted with TRIzol reagent (Vezyme Biotech). mRNA and lncRNA sequencing was performed by Genedenovo.

### Biochemical index assay

Alanine aminotransferase (ALT) and aspartate aminotransferase (AST) kits (Whitman) were used to measure AST and ALT levels in mouse serum. A free fatty acid (FFA) assay kit (Elabscience) was used to detect FFA levels in the serum and liver of the mice. Total triglyceride (TG), total cholesterol (TC), high-density lipoprotein (HDL) or high-density lipoprotein cholesterol (HDL-C) and low-density lipoprotein (LDL) or low-density lipoprotein cholesterol (LDL-C) kits were purchased from Jiancheng and used for the determination of corresponding indicators in mouse serum and liver.

### Oil Red O staining

Liver tissue sections from mice were fixed with 4% paraformaldehyde for 15 min, treated with 60% isopropyl alcohol for 5 min, stained with Oil Red O (Servicebio) for 20 min at 60 °C, decolorized with 60% isopropyl alcohol for 5 min and then stained with hematoxylin at room temperature for 10 min. The slices were sealed with sealing liquid (90% glycerin and 10% ddH_2_O) and observed under a BX-53 microscope (Olympus).

### FA content assay

Liquid chromatography–tandem mass spectrometry (LC–MS/MS) with dinitrophenylhydrazine as the derivatization reagent and quantitative metabolomics analysis were used to determine the content of individual FA components in mouse plasma and liver tissues. Separation was performed using a QTrap 5500 triple quadrupole linear ion trap mass spectrometer (AB Sciex) equipped with an Agilent Poroshell 120 C18 column. Multiple reaction monitoring in electrospray ionization negative mode was used for MS detection. Aqueous 0.5% formic acid (A)–acetonitrile (B) gradient elution was performed at a flow rate of 500 μl/min. The column temperature and sample tray temperature were 40 °C and 4 °C, respectively, and the injection volume was 2.0 μl. Quantification was performed by Workstation Analyst software version 1.6.2 (AB Sciex).

### Peroxidase activity assay

Peroxidase activity was measured in mouse liver tissues and serum using a peroxidase activity assay kit (Sigma-Aldrich). The procedure was conducted as instructed.

### Real-time qPCR

Total RNA from cell or tissue samples was extracted with TRIzol Reagent (Vazyme Biotech). HiScript Q RT SuperMix for qPCR (+gDNA wiper) (Vazyme Biotech) was used to remove DNA and perform reverse transcription. Quantitative polymerase chain reaction (qPCR) was performed using AceQ qPCR SYBR Green Master Mix (Vazyme Biotech) in an Applied Biosystems StepOnePlus real-time PCR system. The relative gene expression was calculated by the ΔΔCT method using 18S as the internal reference gene. The quantitative real-time PCR (qRT‒PCR) sequences of primers used are listed in Table 2.

**Table 2:** Primers used for qRT–PCR analysis.

Primer names	Forward (5′ to 3′)	Reverse (5′ to 3′)
18s	ACGGACCAGAGCGAAAGCAT	TGTCAATCCTGTCCGTGTCC
Gm19656	CTCGGGCTGACTTGACAAA	GTTATTGCAGGCGGGAAACT
Meg3	GTTGTGCTCAGGTTCCACGA	AACGTGTTGTGCGTGAAGTC
C3300001G04Rik	ACGGTAACAGACCCGATCCT	GACTGGCTGACTGGGTTTGA
G730013B0R5ik	TGACGACGGCTCTCCTAGAT	GTTCAGATTCCCTTGGTGGA
A730020M07Rik	CGGACTTTGGGCTACATGAT	ACCAATTCCGAACCTCTGC
Gm13021	GGGTCTGCAAAGTTTCTCCA	CTTCAGCGGCTCAGGAAGT
BC026762	CAGGGCAGTCACCTCAGATT	TTGGCTCTTAGGCTCACAGG
Gm26643	CTTCCGGCGTATTAGTCCAC	CGGCTGTGGGTACAGAATTT
C030034L19Rik	CTTCAGCGGCTCAGGAAGT	GCAGAGGGACTGTCTGTTCG
CT009713.3	TTCCACTCCAGTACCCTTGG	GAGAGATGGCTCCGTGGTTA
A930003O13Rik	ACAGGAACGTGCTTTGACCT	CCCAGCTCTTGAAGTCCATC
Gm26511	GTCAGGTGTCCTCCTTGGTT	GTGTGAACTGCCATGTGTGG
AC132239.1	TCTGGCAGGACCAGTTTACA	GCGCAGAGAACAGCCTTTAT
Gm38832	ACTGCTGCTGAAAGGTGACA	TGTCTGCCACCCAGTCTGTA
Gm30211	AGCCACCAAAGGAAGGAGAT	GGCAGGTGTGAGAGGTGACT
4930479H17Rik	GAGCCACACTCAGAGCAGGT	TTTCCGAACACACTTTCACG
2010015M23Rik	CCCATGTATGCTGTGCATGT	ACAAGGGAAATGGCTCAGTG
Gm38811	AAGCCCATGACAGCCTCTTA	CAGTGCCAATTACCCAGCTC
Gm27000	CAGTCTTTCCAGGACCACCTT	ATCCTCTTTGTCGCTTGTCC
AC160562.1	TTTGAACAGGGTCTCCAAGG	GGCTGTCAGGGAATGTCTTC
AC164092.4	CCAGCCTACCTCTGAGCATC	CCTGCTTGCGTTTCCATTAT
AY036118	CGCTGAGCCAGTCAGTGTAG	GTGCATGGCCGTTCTTAGTT
CT010462.2	AAAGCAATGGAGTTGGCATC	GAGCCAAGAGGATCAAACCA
D630033O11Rik	CAGAAAGAACCAGCCCTTTG	TGGTCCAAGTCCCAGGTTAG
AC160562.2	CGTGTGTGGTGAAGGAAGTG	CACTTGGTGAGTTGGCTCCT
Gm35585	GCAGCAGATGGTGTCTCACA	TGCTGAGTCCTCGGAGTGTA
Ehhadh	ATGATCCGCCTCTGCAATCC	GCTCCACAGATCACTATGGCT
Pparα	TGCAAACTTGGACTTGAACG	GATCAGCATCCCGTCTTTGT
Rxrα	GTGAAAGATGGGATTCTCCTGGC	GTCACGCATCTTAGACACCAGC
Rxrβ	ATCCTGTCCACAGGCATCTCCT	ATCCTGTCCACAGGCATCTCCT
Acc	TGAATCTCACGCGCCTACTATG	ATGACCCTGTTGCCTCCAAAC
Fas	AAGTTGCCCGAGTCAGAGAA	CGTCGAACTTGGAGAGATCC
Srebp1	GGAGCCATGGATTGCACATT	GGAAGTCACTGTCTTGGTTGTTGA
Scd1	TTCTTGCGATACACTCTGGTGC	CGGGATTGAATGTTCTTGTCGT
Fatp1	TGCCACAGATCGGCGAGTTCTA	AGTGGCTCCATCGTGTCCTCAT
Cd36	GGACATTGAGATTCTTTTCCTCTG	GCAAAGGCATTGGCTGGAAGAAC
Cpt1α	GGCATAAACGCAGAGCATTCCTG	CAGTGTCCATCCTCTGAGTAGC
Acox1	GCCATTCGATACAGTGCTGTGAG	CCGAGAAAGTGGAAGGCATAGG
Apob	GCATGAGTATGCCAATGGTCTCC	CTGGTTGCCATCTGAAGCCATG
Mttp	CCAGGAAAGGTTCCTCTATGCC	GACTCTCTGATGTCGTTGCTTGC

### Western blot

Protein extracts were separated on 10–12% sodium dodecyl sulfate polyacrylamide gels and transferred by electroblotting onto polyvinylidene difluoride membranes (Millipore). The membranes were blocked with TBST-prepared 5% skim milk powder for 90 min and then incubated with primary antibodies at 4 °C overnight. The membranes were incubated with the corresponding secondary antibodies at room temperature for 1 h. Protein signals were detected using a Tanon-5200 Multi chemiluminescence imaging system (Tanon). The antibodies used are listed in Table 3.

**Table 3:** Antibodies used.

Name	Citation	Supplier	Cat. no.
β-Actin	WB	Proteintech	66009-1-Ig
CD36	WB	Abclonal	A1470
CPT1α	WB	Proteintech	15184-1-AP
EHHADH	WB, IF	Proteintech	26570-1-AP
GAPDH	WB	Proteintech	60004-1-Ig
Lamin B1	WB	Affinity	AF5161
PPARα	WB	Affinity	AF5301
RXRα	WB	Proteintech	60198-1-Ig
RXRα	Co-IP, ChIP	Abcam	ab125001
RXRβ	WB	Proteintech	14684-1-AP
HRP-conjugated goat anti-mouse IgG (H + L) secondary antibody	WB	Thermo	31430
HRP-conjugated goat anti-rabbit IgG (H + L) secondary antibody	WB	Thermo	31460
Alexa Fluor 647 goat anti-rabbit IgG (H + L)	IF	Jackson ImmunoResearch	111-605-144

IF, immunofluorescence; WB, western blotting.

### Immunofluorescence and Nile Red staining

Tissue sections or cell lysates were fixed with 4% paraformaldehyde and then permeabilized with 1% Triton X-100. The tissues or cells were then blocked with PBS containing 10% goat serum, incubated with 10 µg/ml Nile Red staining solution at 37 °C for 30 min, incubated with an anti-EHHADH antibody at 4 °C overnight and incubated with an Alexa Fluor 647-conjugated goat anti-rabbit IgG (H + L) fluorescent secondary antibody for 1 h. The nuclei were counterstained with DAPI. Fluorescence signals were detected by an FV3000 laser confocal microscope (Olympus).

### RNA FISH

Nucleic-acid-modified targeting Gm35585 oligonucleotide probes and RNA fluorescence in situ hybridization (FISH) kits (GenePharma) were used for RNA FISH of cell migration assays and tissue sections. DAPI staining was used as a counterstain to detect nuclei. Slide imaging was performed using an FV3000 laser confocal microscope (Olympus).

### RNA pulldown assay, silver staining and protein profiling assay

RNA pulldown assays were performed using a magnetic RNA–protein pulldown kit (BersinBio). The RNA pulldown proteins were separated by sodium dodecyl sulfate polyacrylamide gel electrophoresis (SDS–PAGE), and the gels were silver-stained after protein separation using a rapid silver-staining kit (Beyotime). The gels were fixed with fixative, washed with 30% ethanol and water, sensitized by adding silver staining sensitizing solution and washed with water for color development. Then, color development was terminated. The silver-stained gels were analyzed by full protein profiling (Lumingbio).

### ChIP assay

Chromatin immunoprecipitation (ChIP) was performed using a ChIP assay kit (Beyotime) according to the manufacturer’s instructions. Chromatin was broken into 200–1,000 bp DNA fragments using an ultrasonic fragmentation machine. The RXRα antibody was coincubated with protein A + G agarose, and an isotype-matched IgG antibody was added as a negative control. Finally, unlinked and purified DNA fragments and primers for the *Ehhadh* promoter region (F: 5′-TGACCGAGTCCTACAGATGG-3′, R: 5′-GTTGCCAAAGTCCACCAAGT-3′) were used for PCR.

### Co-IP assay

Co-immunoprecipitation (co-IP) was performed using a universal immunoprecipitation kit (Abbkine). Protein A/G magnetic beads were denatured and eluted using 50 µl of 1× SDS–PAGE loading buffer. The samples were heated at 100 °C for 5 min, after which the supernatants were collected.

### Statistics and reproducibility

GraphPad Prism software (GraphPad Software) was used for statistical analysis of the data. The data are expressed as the mean ± s.d. *P* values <0.05 were considered to indicate statistical significance. Representative images of fluorescence staining, Oil Red O staining, silver staining and immunoblotting are shown. Each of these experiments was independently repeated three times. Statistical analyses are justified as appropriate for each figure.

## Results

### Hepatic Gm35585 is downregulated in HFD-fed mice

**Fig. 1:**
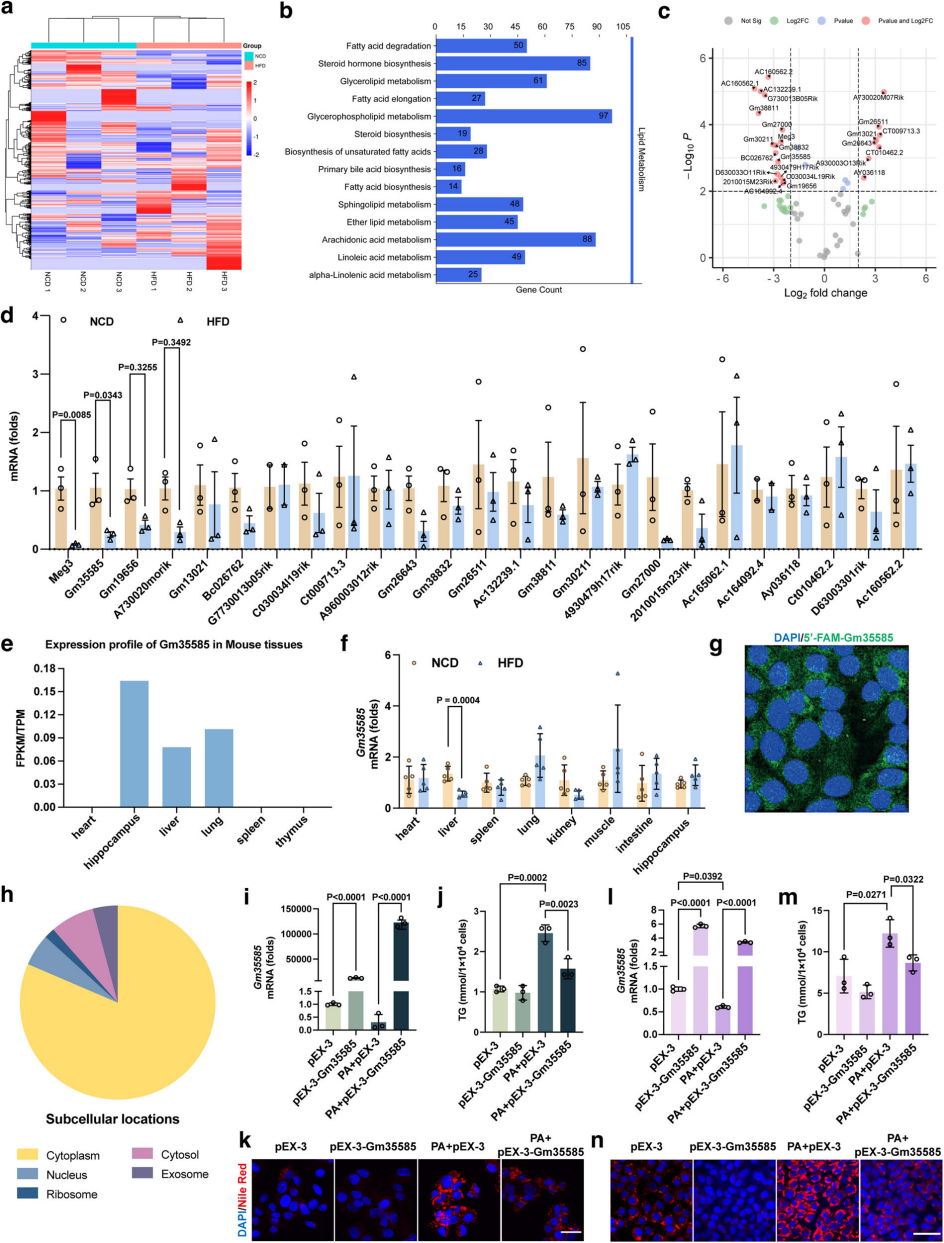
Gm35585 is a novel lncRNA that alleviates lipid accumulation in hepatocytes. **a**, **f** Costaining of liver tissue sections with Nile Red and EHHADH immunofluorescence. Scale bar, 25 μm. **b**, **g** Liver peroxidase activity. Reaction time: 30 min (mean ± s.d., *n* = 5, one-way ANOVA). **c**, **h** Liver total FA contents analyzed by LC–MS/MS (mean ± s.d., *n* = 5, one-way ANOVA). **d**, **i** Heat map of 28 FA contents in the liver. (*P<0.05, **P<0.01, ***P<0.001, NCD+AAV-NC vs HFD+AAV-NC, and NCD+TBG-NC vs HFD+TBG-NC. ^#^*P*<0.05, ^##^*P*<0.01, ^###^*P*<0.001, HFD+AAV-NC vs HFD+AAVGm35585, and HFD+TBG-NC vs HFD+TBG-EHHADH.) **e**, **j** Changes in hepatic SCFA, MCFA and LCFA levels. Information on the 28 FAs is given in Table 4. (**a**–**e** The analysis of Gm35585 overexpression experiments in vivo. **f**–**j** The analysis of EHHADH overexpression experiments in vivo.).

By sequencing the liver lncRNAs of NCD-fed and HFD-fed mice, we identified 1,344 differentially expressed lncRNAs. Cluster analysis revealed significant differences between the groups (Fig. 1a). Pearson correlation coefficient analysis was used to predict the relationships between differentially expressed lncRNAs and all encoded protein mRNAs, and Kyoto Encyclopedia of Genes and Genomes (KEGG) enrichment analysis was performed on the obtained target genes targeting the lipid metabolism signaling pathway. The results showed that the FA degradation pathway was enriched in 50 target genes, 37 of which were significantly differentially expressed (*Cpt1b*, *Cyp4a10*, *Acaa1b*, *Acsl3*, *Acat2*, *Aldh3a2*, *Cyp4a31*, *Acadsb*, *Ehhadh*, *Hadh*, *Acsl5*, *Acsl6*, *Cyp4a14*, *Eci2*, *Aldh9a1*, *Acox3*, *Aldh2*, *Hadhb*, *Acsl4*, *Eci3*, *Acsl1*, *Acadvl*, *Adh5*, *Acadm*, *Cyp4a12b*, *Adh7*, *Gcdh*, *Cpt1c*, *Acox1*, *Cyp4a12a*, *Echs1*, *Acsbg1*, *Adh4*, *Cpt2*, *Adh1*, *Acaa1a* and *Hadha*) (Fig. 1b). Next, we identified the lncRNAs regulated by these 37 target genes (total of 69), among which 25 showed significant differences (*P* > 0.01, |false discovery rate| >4) (Fig. 1c). To validate the sequencing results, we detected the expression of these 25 lncRNAs by qRT‒PCR. We found that the expression of Meg3 and Gm35585 significantly differed in HFD-fed mouse livers (Fig. 1d). In addition to the regulation of lipid metabolism by Meg3 (ref. ^20^), Gm35585 was the most significantly differentially expressed lncRNA, and we chose to further investigate its regulatory function in lipid metabolism.

To investigate the distribution of Gm35585, we searched the NONCODE database (http://www.noncode.org/). The results showed that lncRNA Gm35585 was highly expressed mainly in the liver, lung and hippocampus in mice (Fig. 1e). To confirm these data, we used different organs to determine the tissue distribution of Gm35585. We demonstrated that Gm35585 is expressed in the heart, liver, spleen, lung, kidney, muscle, intestine and hippocampus under physiological conditions. In HFD-fed mice, its expression was upregulated in the lung and muscle and downregulated significantly only in the liver (Fig. 1f). The iLoc-lncRNA database (http://lin-group.cn/server/iLoc-lncRNA/pre.php) predicted that Gm35585 was located mainly in the cytoplasm (Fig. 1h). Moreover, RNA FISH assays and cytoplasmic and nuclear RNA assays revealed that Gm35585 is located mainly in the cytoplasm under physiological conditions (Fig. 1g). These findings were consistent with the database results.

Analysis of Gm35585 conservation using the University of California Santa Cruz (UCSC) database (https://genome-asia.ucsc.edu/cgi-bin/hgGateway) revealed that only a 27-bp homologous region of Gm35585 exists in human (chr4: 113,118,969–113,118,995). To investigate whether Gm35585 has an effect on hepatic lipid accumulation in both mouse and human, pEX-3-Gm355855 plasmid was transfected into AML-12 cells and HepG2 cells. As expected, Gm35585 overexpression upregulated the expression of Gm35585 under PA conditions in HepG2 cells and AML-12 cells (Fig. 1i, l). In HepG2 cells and AML-12 cells, the TG test and Nile Red staining results showed that PA significantly increased the lipid content and promoted lipid accumulation in hepatocytes. However, its lipid-increasing effect was suppressed after Gm35585 overexpression in both cell lines (Fig. 1j–n). In summary, exogenous Gm35585 has a potential role in the treatment of HFD-induced hepatic lipid accumulation in vitro.

### Gm35585 overexpression ameliorates HFD-induced hepatocytic lipid accumulation

**Fig. 2:**
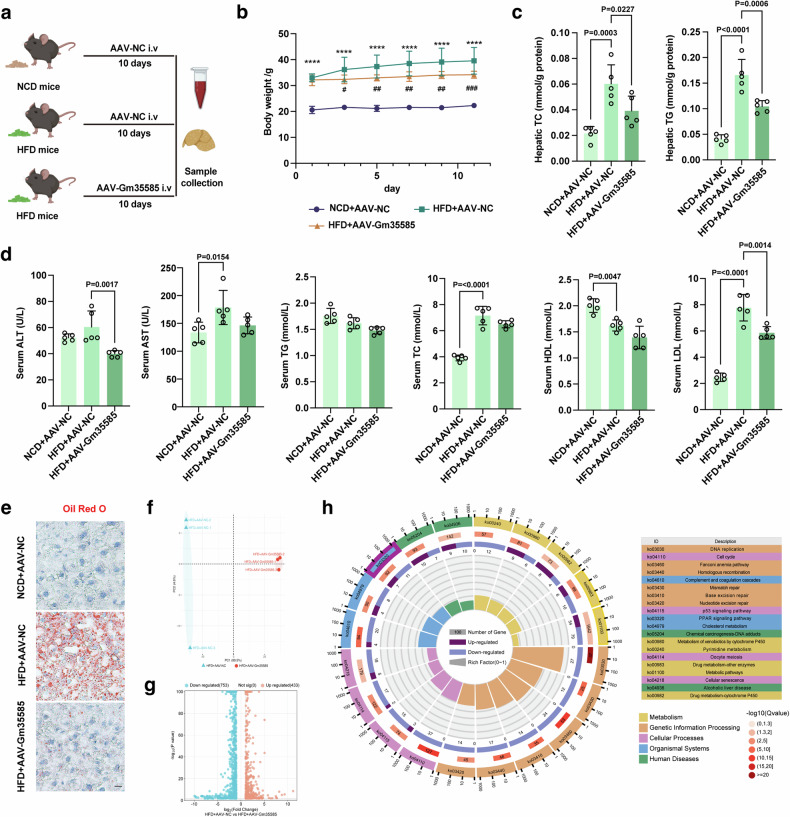
LncRNA Gm35585 reduces HFD-induced hepatocytic lipid accumulation in mice. **a** In vivo experimental flow diagram. NCD mice: C57BL/6J mice; HFD mice: 10 weeks of high-fat feeding C57BL/6J mice. **b** Changes in the body weight of mice within 10 days after AAV-Gm35585 injection (mean ± s.d., *n* = 5, one-way ANOVA; ****P < 0.0001, NCD + AAV-NC versus HFD + AAV-NC; ^#^*P* < 0.05, ^##^*P* < 0.01, ^###^*P* < 0.001, HFD + AAV-NC versus HFD + AAV-Gm35585). **c** Changes in liver TG and TC levels under Gm35585 overexpression condition (mean ± s.d., *n* = 5; one-way ANOVA). **d** Serum biochemical indices included ALT, AST, TG, TC, LDL, and HDL (mean ± s.d., *n* = 5; one-way ANOVA). **e** Oil Red O staining of liver tissue sections indicating changes in lipid droplet content. Scale bar, 50 μm. **f** Differences between groups after Gm35585 overexpression (*n* = 3). **g** The nnumber of upregulated and downregulated genes after Gm35585 overexpression (*n* = 3). **h** KEGG enrichment analysis of RNA sequencing data from HFD + AVV-Gm35585 mouse livers.

Next, we investigated the effect of Gm35585 overexpression on lipid metabolism. The AAV-Gm35585 vector (100 μl per mouse) was delivered to five HFD-fed mice via tail vein injection to establish HFD + AAV-Gm35585 mice, and the AAV-NC vector (100 μl per mouse) was injected into the tail vein of the NCD + AAV-NC and HFD + AAV-NC groups (Fig. 2a). After tail vein injection of the AAV-Gm35585 plasmid into HFD-fed mice for 10 days, Gm35585 expression was significantly upregulated (Supplementary Fig. 1a), and the body weight of HFD + AAV-Gm35585 mice was slightly reduced compared with that of HFD-fed mice (Fig. 2b). Hepatic TG and TC levels were significantly decreased after Gm35585 overexpression (Fig. 2c). The blood biochemistry results revealed a decreasing trend in the serum TG and HDL levels, a significant increase in the TC and LDL levels in HFD-fed mice and a decreasing trend in the ALT, HDL and LDL levels after AAV-Gm35585 administration (Fig. 2d). Liver Oil Red O staining revealed significant accumulation of lipid droplets in the livers of HFD-fed mice, and this accumulation was notably reduced by Gm35585 overexpression (Fig. 2e).

To investigate the mechanism by which Gm35585 reduces lipid accumulation, RNA sequencing analysis was performed. Principal component analysis revealed no overlap between the data from the HFD + AAV-NC and HFD + AAV-Gm35585 groups, indicating a statistically significant difference between the groups (Fig. 2f). A total of 1,186 regulated genes were detected after Gm35585 overexpression; there were 433 upregulated genes and 753 downregulated genes (Fig. 2g). KEGG enrichment analysis indicated that Gm35585-enriched genes were mainly related to metabolic pathways and the PPAR signaling pathway (Ko03320) (Fig. 2h).

### Gm35585 overexpression enhances LCFA metabolism by activating EHHADH

**Fig. 3:**
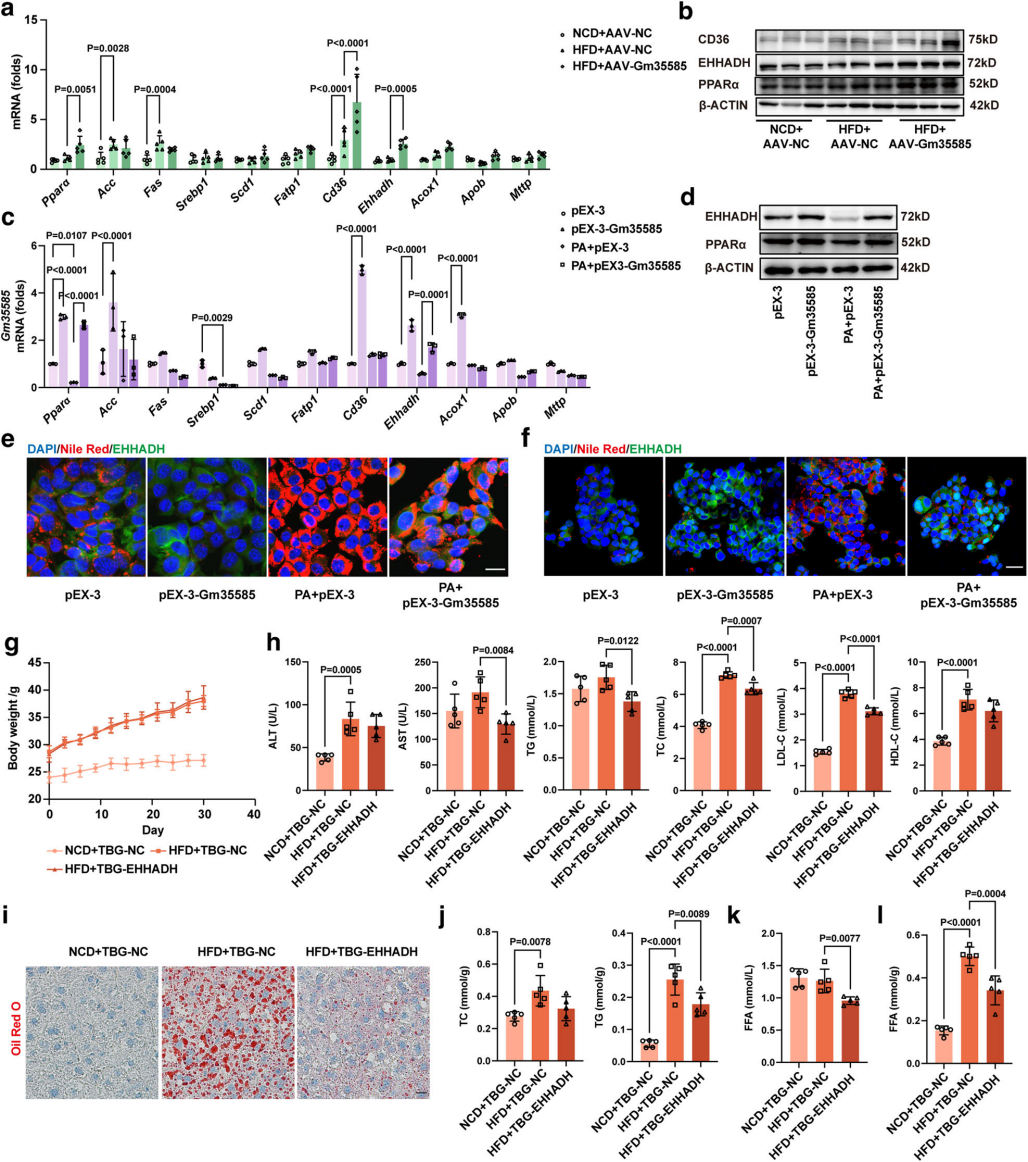
Gm35585 ameliorates hepatic lipid accumulation by regulating EHHADH. **a** The gene expression of *Pparα*, *Acc*, *Fas*, *Srebp1*, *Fatp1*, *Cd36*, *Ehhadh*, *Acox1*, *Apob* and *Mttp* in vivo. The values were normalized to those of the control group (mean ± s.d., *n* = 5; two-way ANOVA). **b** The protein expression of PPARα, EHHADH and CD36 in vivo. **c** The gene expression of *Pparα*, *Acc*, *Fas*, *Srebp1*, *Fatp1*, *Cd36*, *Ehhadh*, *Acox1*, *Apob* and *Mttp* in AML-12 cells. The values were normalized to those of the control group (mean ± s.d., *n* = 3, two-way ANOVA). **d** The protein expression of PPARα and EHHADH in AML-12 cells. **e** Costaining of liver tissue sections with Nile Red and EHHADH immunofluorescence in AML-12 cells. Scale bar, 25 μm. **f** Costaining of liver tissue sections with Nile Red and EHHADH immunofluorescence in HepG2 cells. Scale bar, 50 μm. **g** Changes in the body weight of mice within 1 month after TBG-EHHADH injection. NCD mice: C57BL/6J mice; HFD mice: 5 weeks of high-fat feeding of C57BL/6J mice. **h** Serum biochemical indices included ALT, AST, TG, TC, LDL-C and HDL-C (mean ± s.d., *n* = 5; one-way ANOVA). **i** Oil Red O staining of liver tissue sections indicating changes in lipid droplet content. Scale bar, 50 μm. **j** Changes in liver TG and TC levels (mean ± s.d., *n* = 5; one-way ANOVA). **k** Changes in the serum FFA levels (mean ± s.d., *n* = 5; one-way ANOVA). **l** Changes in the liver FFA levels (mean ± s.d., *n* = 5; one-way ANOVA).

We examined the levels of genes related to the PPAR signaling pathway involved in lipid metabolism. In vivo, Gm35585 overexpression upregulated the expression of PPARα, CD36 and EHHADH at both the mRNA and protein levels compared with those in the HFD group (Fig. 3a, b and Supplementary Fig. 1b). In vitro, compared with those in the PA group, the mRNA and protein levels of PPARα and EHHADH were significantly increased after Gm35585 overexpression (Fig. 3c, d and Supplementary Fig. 1e). Furthermore, *Cpt1α* mRNA expression increased after Gm35585 overexpression, but the protein level did not change (Supplementary Fig. 1c, d). Under PA conditions, Nile Red staining and EHHADH immunofluorescence staining revealed that Gm35585 overexpression upregulated EHHADH protein levels and decreased lipid contents in AML-12 cells and HepG2 cells (Fig. 3e, f).

To explore the function of EHHADH, we induced liver-specific overexpression of the EHHADH adeno-associated virus TBG-EHHADH in HFD-fed mice. The TBG-EHHADH vector (100 μl per mouse) was delivered to five HFD-fed mice by tail vein injection for 1 month to establish HFD + TBG-EHHADH mice, and the TBG-NC vector (100 μl per mouse) was injected into the tail vein of the mice in the NCD + TBG-NC and HFD + TBG-NC groups. After 1 month, mouse livers were sampled to test the EHHADH expression levels to determine the extent of EHHADH overexpression. Compared with those in the HFD group, EHHADH overexpression tended to increase the mRNA level of *Ehhadh* and significantly upregulated the protein level of EHHADH (Supplementary Fig. 1g, h). There were no significant differences in body weight or liver indices between HFD + TBG-NC mice and HFD + TBG-EHHADH mice (Fig. 3g and Supplementary Fig. 1f). The blood biochemistry results showed that there was a significant decrease in the serum AST, TG, TC and LDL levels in HFD + TBG-EHHADH mice (Fig. 3h). Liver Oil Red O staining revealed substantial accumulation of lipid droplets in the livers of HFD-fed mice, and this accumulation was alleviated by EHHADH overexpression (Fig. 3i). Liver TC and TG levels were notably increased in HFD-fed mice but decreased after EHHADH overexpression (Fig. 3j). In addition, the hepatic FFA content was increased in HFD-fed mice, and after EHHADH overexpression, both the serum and hepatic FFA contents in HFD-fed mice were significantly reduced (Fig. 3k, l).

**Fig. 4:**
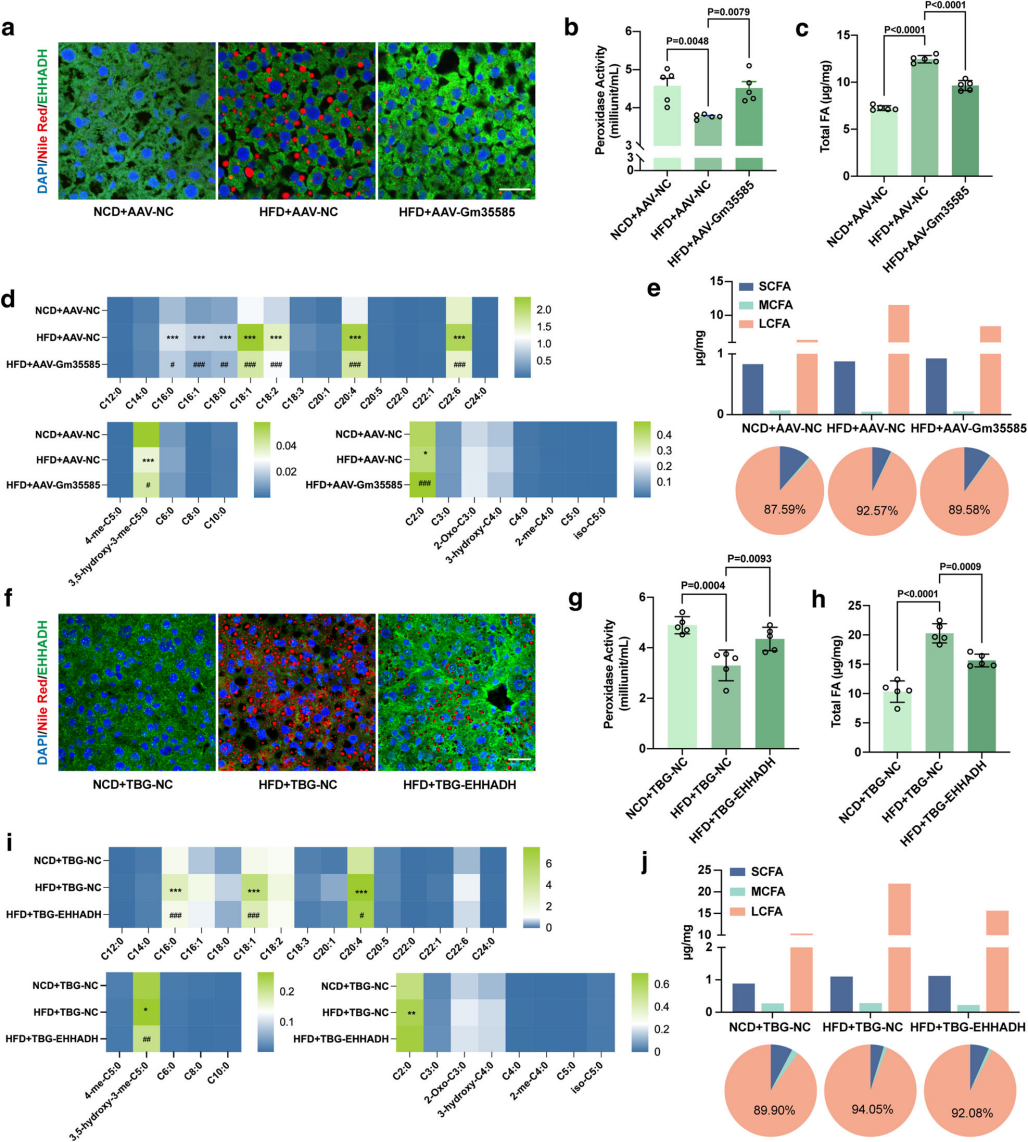
Gm35585 promotes oxidation of LCFAs contents by activating peroxidase EHHADH. **a**, **f** Costaining of liver tissue sections with Nile Red and EHHADH immunofluorescence. Scale bar, 25 μm. **b**, **g** Liver peroxidase activity. Reaction time: 30 min (mean ± s.d., *n* = 5, one-way ANOVA). **c**, **h** Liver total FA contents analyzed by LC–MS/MS (mean ± s.d., *n* = 5, one-way ANOVA). **d**, **i** Heat map of 28 FA contents in the liver. *P < 0.05, **P < 0.01, ***P < 0.001, NCD+AAV-NC *vs* HFD+AAV-NC, and NCD+TBG-NC *vs* HFD+TBG-NC. ^#^*P* < 0.05, ^##^*P* < 0.01, ^###^*P* < 0.001, HFD+AAV-NC *vs* HFD+AAV-Gm35585, and HFD+TBG-NC *vs* HFD+TBG-EHHADH. **e**, **j** Changes in hepatic SCFA, MCFA and LCFA levels. Information on the 28 FAs is given in Table 4. (**a**–**e** The analysis of Gm35585 overexpression experiments in vivo. **f**–**j** The analysis of EHHADH overexpression experiments in vivo).

Next, we explored the relationship between EHHADH and the metabolism of LCFAs. Nile Red staining and EHHADH immunofluorescence costaining were performed on mouse liver sections. Compared with those in the HFD group, the lipid content of the liver in the Gm35585- or EHHADH-overexpressing group decreased, while that of the EHHADH protein increased (Fig. 4a, f). EHHADH is an important protein for the FA β-oxidation of peroxisomal enzymes. The peroxisome is an important site for FA oxidation. Therefore, in the peroxidase activity assay, HFD consumption notably decreased liver peroxidase activity, and AAV-Gm35585 or TBG-EHHADH administration attenuated this effect, thereby significantly increasing liver peroxidase activity (Fig. 4b, g). Unexpectedly, the changes in serum peroxidase activity were opposite to those in the liver (Supplementary Figs. 2a and 3a).

Next, we performed LC–MS/MS to detect the profile of 28 types of FA in the liver and serum. Clearly, the total FA level was highly increased in the livers of HFD-fed mice, but the FA level decreased under conditions of Gm35585 or EHHADH overexpression (Fig. 4c, h). Serum total FA levels changed slightly (Supplementary Figs. 2b and 3b). Furthermore, we compared the individual FA content of the liver and serum. Under Gm35585 overexpression conditions, in HFD-fed mice, only the hepatic acetic acid (C2:0) content of short-chain fatty acids (SCFAs) and the mevalonic acid (3,5-hydroxy-3-me-C5:0) content of MCFAs changed markedly. For LCFAs, hepatic PA (C_16:0_), palmitoleic acid (C_16:1_), stearic acid (C_18:0_), oleic acid (C_18:1_), linolenic acid (C_18:2_), arachidonic acid (C_20:4_) and docosahexaenoic acid (C_22:6_) contents were significantly increased under HFD conditions, and Gm35585 overexpression greatly decreased the contents of these seven LCFAs (Fig. 4d). Similarly, under EHHADH overexpression conditions, the SCFAs content did not notably change, and only the MVA content was reduced in the liver of HFD-fed MCFAs. In LCFAs, hepatic C16:0, C18:1 and C20:4 contents decreased after EHHADH overexpression (Fig. 4i). FA content analysis revealed that hepatic LCFAs were significantly decreased in HFD-fed mice overexpressing Gm35585 or EHHADH, but the SCFA and MCFA contents barely changed (Fig. 4e, j). Neither Gm35585 nor EHHADH overexpression significantly affected the levels of individual FAs in the serum (Supplementary Figs. 2c–e and 3c–e). Together, these data confirmed that lncRNA Gm35585 decreased hepatic LCFA levels, which might be related to peroxisomal EHHADH activation.

### Gm35585 activates EHHADH via the PPARα signaling pathway

**Fig. 5:**
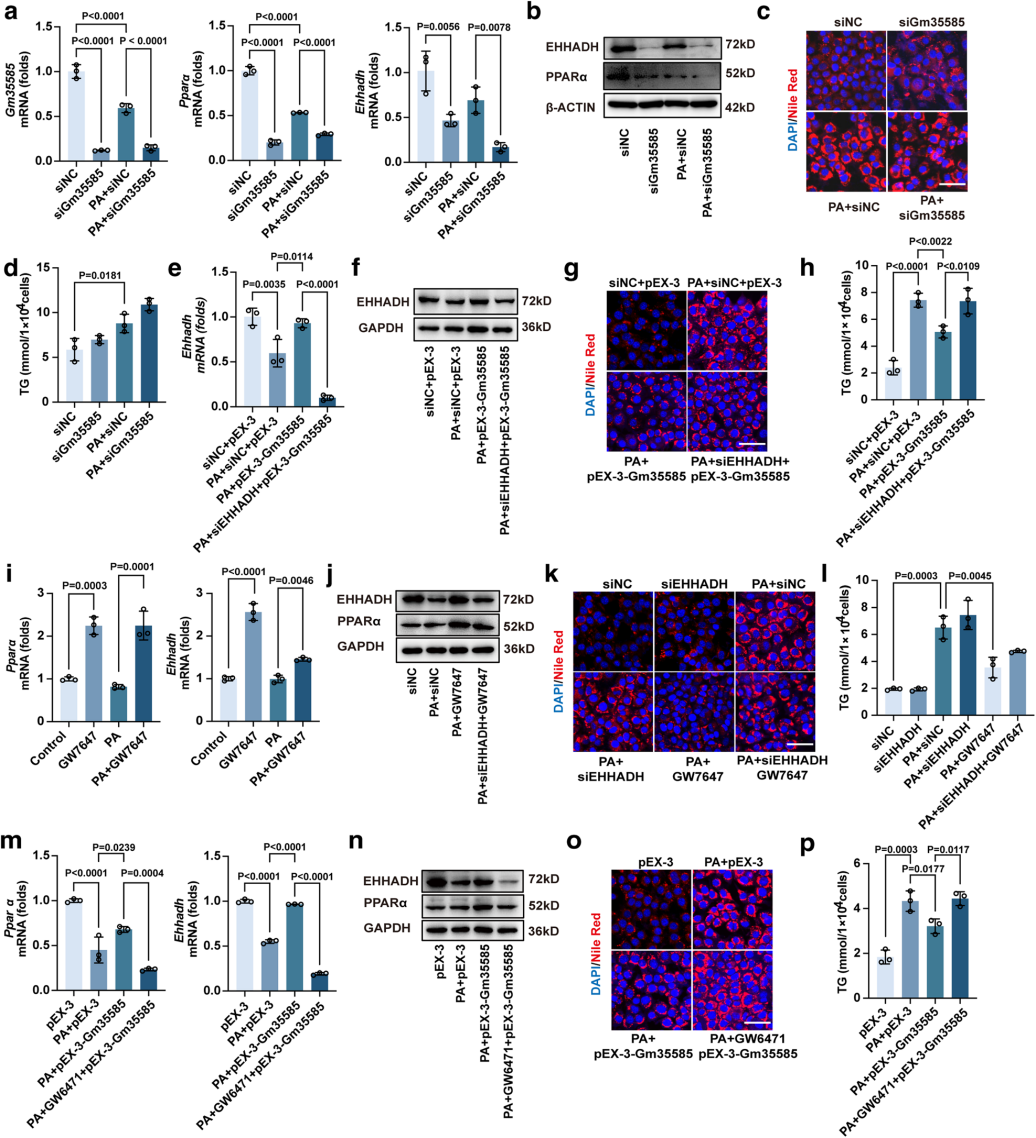
Gm35585 activates EHHADH via the PPARα signaling pathway. **a** mRNA expression levels of Gm35585, Pparα and Ehhadh with PA and siGm35585 treatment in AML-12 cells. The values were normalized to those of the control group (mean ± s.d., *n* = 3, one-way ANOVA). **b**, **j**, **n** The protein expression of PPARα and EHHADH in AML-12 cells. **c**, **g**, **k**, **o** Nile Red staining of AML-12 cells to test changes in lipid content. Scale bar, 50 μm. **d**, **h**, **l**, **p** Changes in TG levels in AML-12 cells (mean ± s.d., *n* = 3, one-way ANOVA). **e** mRNA expression levels of Ehhadh with PA, siEHHADH and pEX-3-Gm35585 treatment in AML-12 cells. The values were normalized to those of the control group (mean ± s.d., *n* = 3, one-way ANOVA). **f** The protein expression of EHHADH in AML-12 cells. **i** mRNA expression levels of Pparα and Ehhadh with PA and GW7647 treatment in AML-12 cells. The values were normalized to those of the control group (mean ± s.d., *n* = 3, one-way ANOVA). **m** mRNA expression levels of Pparα and Ehhadh with PA, GW6471 and pEX-3-Gm35585 treatment in AML-12 cells. The values were normalized to those of the control group (mean ± s.d., *n* = 3, one-way ANOVA). (**b**–**d** The analysis in AML-12 cells with PA and siGm35585 treatment. **g**, **h** The analysis in AML-12 cells with PA, siEHHADH, and pEX-3-Gm35585 treatment. **j**–**l** The analysis in AML-12 cells with PA, siEHHADH, and GW7646 treatment. **n**–**p** The analysis in AML-12 cells with PA, GW6471, and pEX-3-Gm35585 treatment.).

Next, we investigated the upstream–downstream relationships between Gm35585, PPARα and EHHADH. siRNA was used to silence Gm35585 in AML-12 cells. In the presence of PA, siGm35585 decreased the expression of Gm35585, PPARα and EHHADH (Fig. 5a, b and Supplementary Fig. 4a). Nile Red staining and TG analysis indicated that the lipid content increased slightly after Gm35585 silencing (Fig. 5c, d). However, pEX-Gm35585 upregulated the expression of EHHADH, but the EHHADH expression level decreased after siRNA silencing of EHHADH (Fig. 5e, f and Supplementary Fig. 4b). siEHHADH reversed the hypolipidemic effect of pEX-3-Gm35585, leading to elevated lipid content (Fig. 5g, h). These findings indicate that Gm35585 ameliorated lipid accumulation by upregulating the expression of EHHADH.

PPARα is a ligand-dependent nuclear transcription factor, and in the presence of a ligand, PPARα strongly increases EHHADH expression levels^21^. To verify the effect of PPARα on the activation of EHHADH, AML-12 cells were treated with GW7647 (a PPARα-specific agonist) for 24 h. GW7647 significantly increased *Pparα* and *Ehhadh* mRNA expression in the PA group (Fig. 5i). After treatment with siEHHADH, the EHHADH protein level did not increase after GW7647 administration (Fig. 5j and Supplementary Fig. 4c). Then, Nile Red staining and TG analysis were performed to detect changes in lipid content. Lipid levels were not notably changed after siEHHADH administration but were significantly decreased by GW7647 treatment. However, when siEHHADH and GW7647 were used in combination, the lipid level significantly increased compared with that under GW7647 treatment alone (Fig. 5k, l). These data show that PPARα regulates the involvement of EHHADH in lipid metabolism.

To determine whether Gm35585 influences PPARα, AML-12 cells were treated with the PPARα inhibitor GW6471 for 24 h before pEX-3-Gm35585 administration. The levels of PPARα and EHHADH both increased after Gm35585 overexpression but decreased after GW6471 administration (Fig. 5m, n and Supplementary Fig. 4d). Nile Red staining and TG analysis also revealed that GW6471 reversed the lipid-lowering effect of pEX-3-Gm35585 and the increase in lipid content (Fig. 5o, p). Overall, Gm35585 regulates lipid accumulation, potentially by activating the PPARα–EHHADH signaling pathway.

### Gm35585 induces PPARα/RXRα heterodimer formation to promote EHHADH transcription

**Fig. 6:**
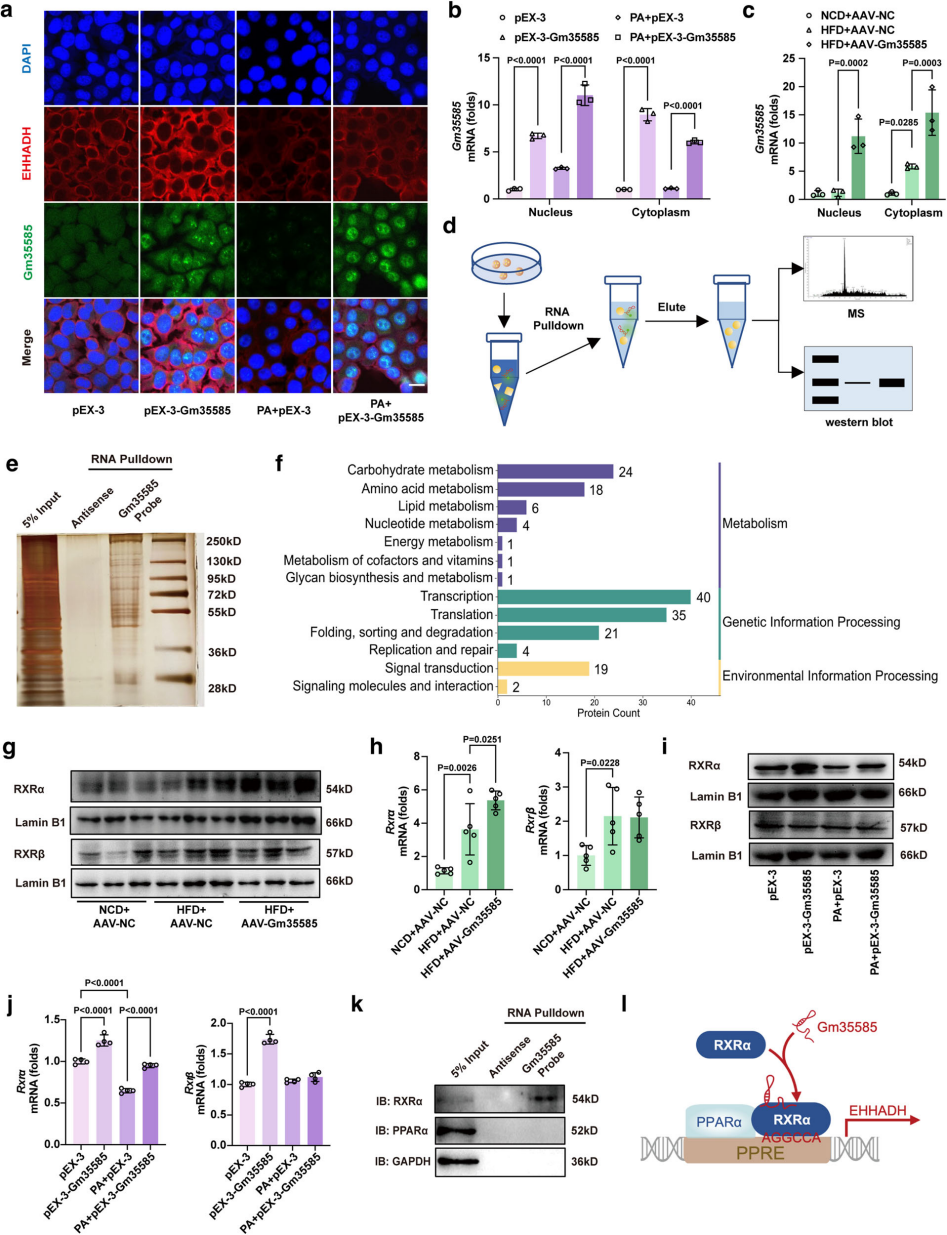
Gm35585 rescues RXRα to promote the formation of PPARα/RXRα heterodimer. **a** Gm35585 RNA FISH and EHHADH immunofluorescence costaining under PA and pEX-3-Gm35585 treatment in AML-12 cells. Scale bar, 25 μm. **b** Gm35585 expression levels in the nucleus and cytoplasm of AML-12 cells. The values were normalized to those of the control group (mean ± s.d., *n* = 3, two-way ANOVA). **c** Gm35585 expression in the nucleus and cytoplasm in the liver. The values were normalized to those of the control group (mean ± s.d., *n* = 3, two-way ANOVA). **d** RNA pulldown assay flowchart in AML-12 cells. **e** Silver staining followed by an RNA pulldown assay in AML-12 cells. A U-biotin-labeled Gm35585 probe was used to pull down proteins. **f** KEGG enrichment analysis of the RNA pulldown protein profile. **g** Protein expression levels of RXRα and RXRβ in Gm35585 overexpression mouse liver. **h** mRNA expression levels of *Rxrα* and *Rxrβ* in Gm35585 overexpression mouse liver. The values were normalized to those of the control group (mean ± s.d., *n* = 5; one-way ANOVA). **i** Protein expression levels of RXRα and RXRβ in AML-12 cells. **j** mRNA expression levels of *Rxrα* and *Rxrβ* in AML-12 cells. The values were normalized to those of the control group (mean ± s.d., *n* = 4, one-way ANOVA). **k** Western blot analysis of RNA pulldown proteins. **l** Mechanistic diagram of Gm35585 binding to RXRα.

We further investigated the interaction mechanism of Gm35585, PPARα and EHHADH. The distribution of Gm35585 and EHHADH was detected by costaining with RNA FISH and immunofluorescence. Physiologically, both Gm35585 mRNA and the EHHADH protein were distributed in the cytoplasm. Interestingly, after pEX-3-Gm35585 treatment, EHHADH protein levels increased in the cytoplasm, but Gm35585 levels increased in the nucleus (Fig. 6a). Moreover, after cytoplasmic and nuclear RNA isolation and extraction, Gm35585 mRNA expression in the cytoplasm and nucleus was measured by qRT–PCR. Gm35585 expression levels were elevated in both the nucleus and cytoplasm under AdGm35585 treatment in vitro and in vivo (Fig. 6b, c). Based on these results, we hypothesized that Gm35585 functions as a chaperone that guides transcription factor nuclear translocation, indicating transcriptional regulation.

To identify the protein that binds to Gm35585, an RNA pulldown assay was performed in vitro (Fig. 6d). After pEX-3-Gm35585 treatment, a U-biotin Gm35585 probe was used to pull down proteins, and an antisense Gm35585 probe was used as the negative control. Sliver staining revealed the proteins that bound to Gm35585 (Fig. 6e). Then, the silver-stained gels were excised for LC–MS, and 377 proteins were detected (Supplementary Fig. 5a). KEGG enrichment indicated that 40 proteins bound to Gm35585 were enriched in transcriptional regulatory pathways (Fig. 6f). Among these 40 proteins, RXR, a ligand-dependent transcription factor that interacts with PPAR^21^, may be associated with the involvement of Gm35585 in lipid metabolism.

There are three isoforms of RXRs in mice: RXRα, RXRβ and RXRγ. RXRα is found in the kidney, liver and intestine; RXRβ is present in almost every tissue, and RXRγ is present mainly in the pituitary gland, brain and muscles^22^. Thus, we examined the levels of RXRα and RXRβ in vitro and in vivo. Interestingly, after AAV-Gm35585 delivery, RXRα expression levels increased significantly, but RXRβ expression levels did not change (Fig. 6g,h and Supplementary Fig. 5b). Similarly, in an in vitro assay, RXRα was highly expressed after pEX-3-Gm35585 treatment, but RXRβ expression was not significantly different (Fig. 6i, j and Supplementary Fig. 5c). Furthermore, we verified the interaction between Gm35585 and RXRα. The results showed that Gm35585 bound to RXRα but not PPARα (Fig. 6k). Therefore, we hypothesized that Gm35585 probably binds to RXRα to form PPARα/RXRα heterodimers to promote lipid metabolism (Fig. 6l).

### Activation of the Gm35585–PPARα/RXRα–EHHADH axis alleviates hepatocytic lipid accumulation

**Fig. 7:**
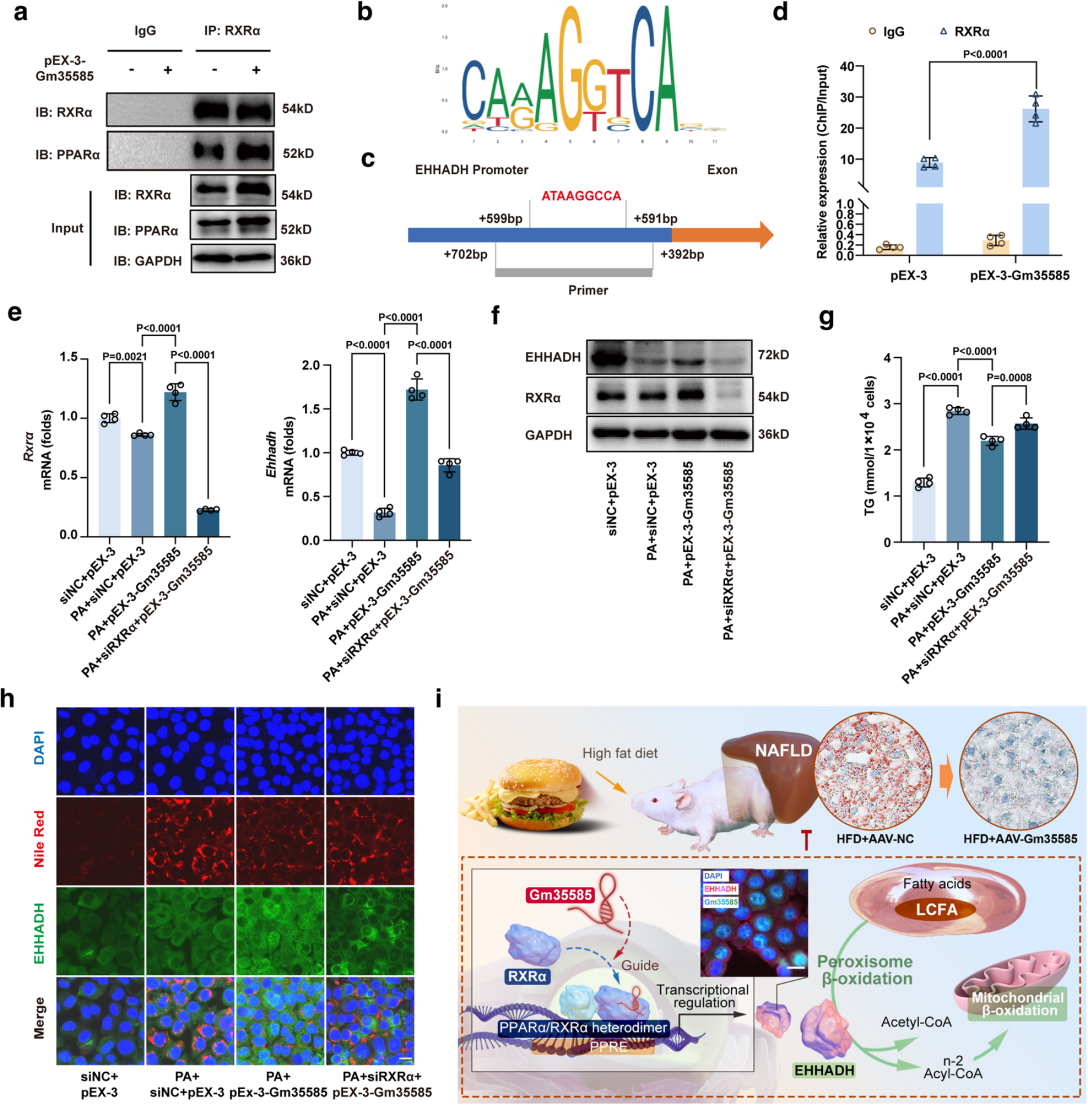
Gm35585 transregulates EHHADH expression by binding PPARα/RXRα heterodimers. **a** Western blotting assay followed by co-IP assay under IgG and RXRα antibody treatment in AML-12 cells. **b** The JASPAR database shows the sequence of the PPARα/RXRα transcription factor DAN, which binds to the EHHADH promoter. **c** The position of the ChIP primer in the EHHADH promoter; the red font represents a DNA sequence similar to that in (**b**). **d** A ChIP assay combined with RT‒qPCR was performed using the IgG and RXRα antibodies in AML-12 cells (mean ± s.d., *n* = 4, *t* test). **e** mRNA expression levels of *Rxrα* and *Ehhadh* with PA, siRXRα and pEX-3-Gm35585 treatment in AML-12 cells. The values were normalized to those of the control group (mean ± s.d., *n* = 4, one-way ANOVA). **f** Protein expression levels of RXRα and EHHADH in AML-12 cells. **g** Changes in TG levels in AML-12 cells (mean ± s.d., *n* = 4, one-way ANOVA). **h** Nile Red and EHHADH immunofluorescence costaining in AML-12 cells. Scale bar, 25 μm. **i** Total mechanism chart.

RXRα is a ligand-dependent transcription factor that interacts with PPARα to form PPARα/RXRα heterodimers and binds to DNA sequences to form PPAR response elements that control the transcription of downstream target genes^21^. Thus, we used an anti-RXRα antibody for the co-IP assay to verify the binding of PPARα to RXRα. As expected, compared with pEX-3 treatment, pEX-3-Gm35585 treatment increased PPARα binding to RXRα (Fig. 7a). Based on the RNA–protein–DNA ternary mode, we further verified whether RXRα bound to the EHHADH promoter. The JASPAR database (https://jaspar.genereg.net) was used to predict the transcription factors that bind to the EHHADH promoter, and a sequence involved in PPARα/RXRα was predicted (from +599 bp to +591 bp) (Fig. 7b). Thus, we designed a primer sequence for the ChIP assay to verify the binding of RXRα to the EHHADH promoter. Within the range of sequences covered by the primer, there was a sequence that was highly matched to that in Fig. 6b (Fig. 7c). ChIP assays indicated that RXRα bound to the EHHADH promoter and that the binding efficiency of both was significantly increased after Gm35585 overexpression (Fig. 7d).

To determine the effect of RXRα on EHHADH and lipids, we used siRNA to silence RXRα expression in vitro. After siRXRα silencing, Gm35585 overexpression no longer upregulated EHHADH mRNA or protein expression in the presence or absence of PA (Fig. 7e, f and Supplementary Fig. 6a–d). Then, we examined the changes in lipid content. TG levels were significantly increased after PA treatment but decreased after pEX-3-Gm35585 administration. However, these parameters were significantly increased via siRXRα silencing (Fig. 7g). Nile Red and EHHADH immunofluorescence costaining was also performed. Both the lipid content and EHHADH protein fluorescence intensity under PA conditions increased after pEX-3-Gm35585 administration, but these effects were reversed by siRXRα silencing, leading to an increase in lipid content and a decrease in EHHADH protein expression (Fig. 7h). Together, these data support that Gm35585 binds to RXRα, increases the formation of PPARα/RXRα heterodimers, promotes RXRα binding to the EHHADH promoter and upregulates EHHADH expression, thereby activating the peroxisome β-oxidation pathway to reduce the LCFA content and ameliorate hepatocyte lipid accumulation (Fig. 7i).

## Discussion

In recent years, lncRNAs, such as H19^23^, NEAT1^24^ and HULC^25^, have been shown to play essential roles in MASLD. Here, we identified a novel lncRNA, Gm35585, that is related to hepatic lipid metabolism. Although Gm35585 was widely distributed, its expression levels were reduced only in the liver after HFD feeding (Fig. 1f). The results demonstrated that overexpression of Gm35585 is important for lipid regulation both in vivo and in vitro, as it serves as a positive factor in the control of lipid metabolism, especially LCFA metabolism. This result indicated that the liver might be a specific target tissue of Gm35585 in the regulation of lipid metabolism. We performed a conserved analysis of Gm35585 through the UCSC database. It showed that only a 27-bp homologous region of Gm35585 exists in human (chr4: 113,118,969–113,118,995). Due to its short length, this human sequence fragment was supposed to have no role in guiding RXRα-mediated transcriptional regulation. However, in our study, Gm35585 also had therapeutic effects on HepG2 cells in a HFD model (Fig. 1j, k). The results indicated that exogenous administration of Gm35585 might be a treatment for diet-induced fatty liver in human. Gm35585 expression was downregulated under HFD conditions, which implied that HFD could affect transcription of Gm35585, but the mechanism remains unexplored. In a previous study, we demonstrated that tamoxifen reduced HFD mice hepatic lipid accumulation via activating EHHADH^26^. However, it is not clear whether tamoxifen affects Gm35585 transcription; the endogenous regulators of Gm35585 need to be further examined.

LCFA and very long chain fatty acid (VLCFA) act as substrates for peroxisomal β-oxidation. Under HFD conditions, peroxisome activity is reduced, and excess LCFA and VLCFA cannot be properly oxidized, which leads to hepatic steatosis^15^. In this study, we found that the contents of oleic acid, linolenic acid, arachidonic acid and docosahexaenoic acid changed significantly in the HFD group (Fig. 4d, f). Their increased levels play an important role in disorders of lipid metabolism. Excess oleic acid induces the excessive accumulation of hepatic reactive oxygen species and oxidative damage^27^ or induces fat abnormalities in hepatocytes, causing toxic damage^28^. A HFD rich in linolenic acid and arachidonic acid may induce oxidative stress and hepatocyte death, promoting the liver inflammatory response, steatosis and liver dysfunction^29,30^. Moderate amounts of docosahexaenoic acid have beneficial effects on brain function, antitumor activity and lipid and glucose metabolism^31^, but under HFD conditions, excess docosahexaenoic acid may exacerbate hepatocyte apoptosis and trigger susceptibility to lipid peroxidation^32^. These reports support that LCFA and VLCFA are important factors in HFD-induced liver injury. Targeting the metabolic enzymes of LCFA and VLCFA could be a suitable therapeutic strategy for MASLD.

EHHADH is part of the peroxisomal FA β-oxidation pathway and is highly induced via PPARα^33^. EHHADH deficiency inhibits dicarboxylic acid β-oxidation and hepatic cholesterol biosynthesis^34^. Moreover, EHHADH is related to various cancer types, including osteosarcoma^35^, bladder cancer^36^, ovarian cancer^37^ and hepatoblastoma^38^. In our study, we found that high EHHADH expression was closely associated with the alleviation of HFD-induced MASLD. High EHHADH expression still upregulated hepatic peroxidase activity and reduced the LCFA content in the liver (Fig. 4g,i). However, virtual docking screening indicated that the crystal structure of EHHADH was not suitable for direct targeting of small molecule compounds (Supplementary Fig. 4e). Transcriptional activation may be the only pathway for EHHADH regulation. We revealed that Gm35585 overexpression upregulated the expression of EHHADH, increased liver peroxidase activity and reduced FA content. Furthermore, after PPARα and EHHADH suppression, Gm35585 overexpression no longer reduced the lipid content. These results indicated that Gm35585 is a potential small nucleic acid drug that can target EHHADH to enhance FA β-oxidation.

PPARs are critical regulators of FA metabolism, glucose metabolism, inflammation and fibrogenesis. Among the PPAR subtypes, PPARα is highly expressed in hepatocytes^39^. In the hepatic lipid metabolic pathway, FA transport, mitochondrial and peroxisomal β-oxidation and lipolysis are mediated mainly by PPARα, resulting in a reduction in TG accumulation^40^. In this study, we found that, among the PPAR-related differential expression genes after the treatment of Gm35585 under HFD condition, the change in EHHADH was consistent in vivo and in vitro (Fig. 3a–d). Meanwhile, the expression CD36 could not be changed by Gm35585 in vitro under PA condition. The reason for this phenomenon needs further verification. We hypothesized that EHHADH is an important regulatory site for Gm35585 in the PPAR pathway. Moreover, administration of the PPARα agonist GW7647 reduced hepatocyte lipid levels. However, the lipid content was elevated after silencing EHHADH, and the PPARα inhibitor GW6471 reversed the upregulation of Gm35585 by EHHADH (Fig. 5i–p). These results indicated that, although PPARα has a wide range of metabolic regulatory effects, EHHADH is a main downstream regulator of PPARα in lipid metabolism in hepatocytes.

As a member of the nuclear receptor superfamily, RXR regulates gene expression^41^. RXR heterodimerizes with nuclear receptors such as PPAR, LXR and FXR to form permissive heterodimers that affect the transcription of target genes and regulate the metabolic response of lipids or bile acids^42,43^. Among the RXR heterodimerization partners, PPAR is one of the most studied. It recognizes and binds direct repeat (DR)1 DNA sequences (5′-AGGTCA-3′) as response elements, and during DNA binding, the heterodimer binds to DNA sequences through the zinc finger motif of the DNA binding domain of each monomer^44–46^. Here, we verified that Gm35585 did not enter the nucleus under physiological conditions, but exogenous Gm35585 facilitated the binding of Gm35585 to RXRα and directs Gm35585 into the nucleus (Fig. 6a, k). Furthermore, under the condition of Gm35585 overexpression, PPARα bound RXRα to form a heterodimer that translocated to the EHHADH promoter region (Fig. 7a–d). There is a DNA sequence (5′-AGGCCA-3′) in the EHHADH promoter region (from +599 bp to +591 bp) that is highly similar to the DR DNA sequence. However, a wider range of primers were used for the ChIP assay. Whether this sequence is the binding site of RXRα for EHHADH needs further confirmation. From the RNA pulldown assay, we found that Gm35585 bound to RXRα directly instead of to PPARα, which suggests the important role of the RXRα/PPARα heterodimer in PPARα-related lipid metabolism. In addition, the expression of RXRα was also increased by the administration of Gm35585, indicating that the effect of Gm35585 on EHHADH was based on both the upregulation and guidance of RXRα. Moreover, it is necessary to conduct in-depth studies on the combination of Gm35585 and RXRα, which might provide a new target for MASLD drug development.

Take together, this Article shows that Gm35585 guides RXRα nuclear translocation to form the RXRα/PPARα heterodimer. This heterodimer activates EHHADH transcription to increase peroxisome activity and metabolizes excess LCFA and VLCFA over time, thereby improving HFD-induced fatty liver. This study revealed for the first time that Gm35585 regulates hepatocytic lipid metabolism and provides a new therapeutic strategy for MASLD.

**Table 4:** Twenty-eight types of FA.

	Designation	Abbreviation
C2:0	Acetic acid	AA
C3:0	Propionic acid	PPA
C4:0	Butyric acid	BA
C5:0	Valeric acid	VA
C6:0	Hexanoic acid	HXA
C8:0	Octanoic aicd	OTA
C10:0	Decanoic acid	DA
*iso*-C5:0	*iso*-Valeric acid	iso-VA
2-Oxo-C3:0	Pyruvic acid	PRA
2-Hydroxy-C4:0	β-Hydroxybutyrate	HDBT
2-me-C4:0	2-Methylbutyric acid	2-me-BA
4-me-C5:0	4-Methylvaleric acid	4-me-VA
3,5-Hydroxy-3-m3-C5:0	Mevalonic acid	MVA
C12:0	Dodecanoic acid	DDA
C14:0	Myristic acid	MRA
C16:0	Palmitic acid	PA
C16:1	Palmitoleic acid	PMA
C18:0	Stearic acid	SA
C18:1	Oleic acid	OA
C18:2	Linoletic acid	LLA
C18:3	Linolenic acid	LA
C20:1	Eicosenoic acid	ESA
C20:4	Arachidonic acid	ARA
20:05	Eicosapentaenoic acid	EPA
C22:0	Behenic acid	BHA
22:01	Erucic acid	EA
C22:6	Docosahexaenoic acid	DHA
C24:0	Lignoceric acid	LNA

## Supplementary information

Supplementary Information
