# Supplementary material for: LncRNA Gm35585 transcriptionally activates the peroxidase EHHADH against diet-induced fatty liver
Source: Exp Mol Med. 2025 Mar 13;57(3):652–66. doi: 10.1038/s12276-025-01420-5 (PMC11958773; doi:10.1038/s12276-025-01420-5)
Supplement: Supplementary file 1 — Supplementary Information [file 12276_2025_1420_MOESM1_ESM.pdf]

Supplementary Figures

Supplementary Fig. 1

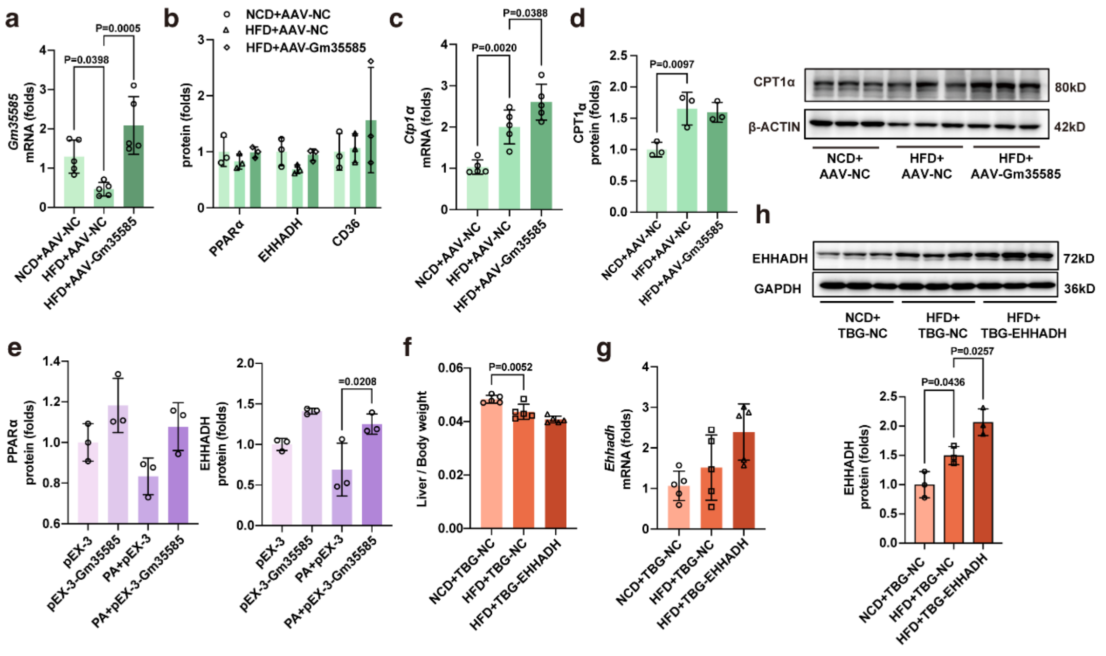

Supplementary Fig. 1. mRNA and protein expression levels of mice.

a Detection of Gm35585 expression changes in liver tissues by qRT–PCR. Values were normalized to the control group. (mean ± SD, *n* = 5, One-way ANOVA analysis). **b** Protein expression levels of PPARα, EHHADH, and CD36. (mean ± SD, *n* = 3, Two-way ANOVA analysis). **c** *Cpt1α* mRNA expression level. (mean ± SD, *n* = 5, One-way ANOVA analysis). **d** CPT1α protein expression level. (mean ± SD, *n* = 3, One-way ANOVA analysis). **e** Protein expression levels of PPARα, EHHADH. (mean ± SD, *n* = 3, Two-way ANOVA analysis). **f** Liver/body weight factor. (mean ± SD, *n* = 5, One-way ANOVA analysis). **g** *Ehhadh* mRNA expression level. (mean ± SD, *n* = 5, One-way ANOVA analysis). **h** EHHADH protein expression level. (mean ± SD, *n* = 3, One-way ANOVA analysis).

Supplementary Fig. 2

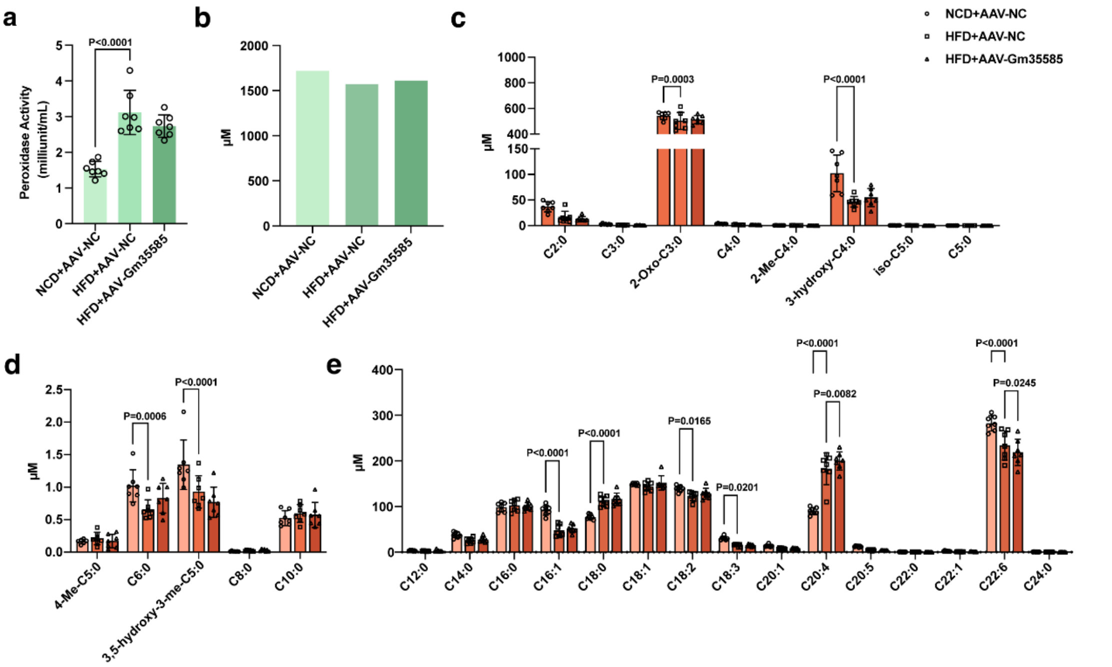

Supplementary Fig. 2. Serum fatty acids content and peroxidase activity in Gm35585 overexpression mice.

a Peroxidase activity. (mean ± SD, *n* = 7, One-way ANOVA analysis). **b** Total fatty acids contents. **c** SCFAs contents. (mean ± SD, *n* = 7, Two-way ANOVA analysis). **d** MCFAs contents. (mean ± SD, *n* = 7, Two-way ANOVA analysis). **e** LCFAs contents. (mean ± SD, *n* = 7, Two-way ANOVA analysis).

Supplementary Fig. 3

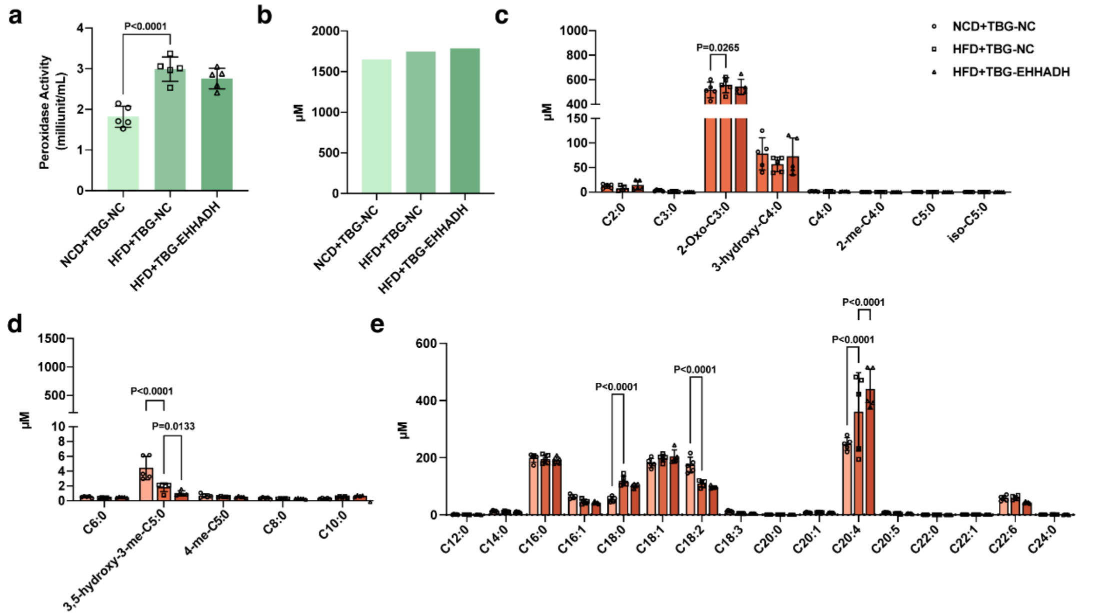

Supplementary Fig. 3. Serum fatty acids content and peroxidase activity in EHHADH overexpression mice.

a Peroxidase activity. (mean ± SD, *n* = 5, One-way ANOVA analysis). **b** Total fatty acids contents. **c** SCFAs contents. (mean ± SD, *n* = 5, Two-way ANOVA analysis). **d** MCFAs contents. (mean ± SD, *n* = 5, Two-way ANOVA analysis). **e** LCFAs contents. (mean ± SD, *n* = 5, Two-way ANOVA analysis).

Supplementary Fig. 4

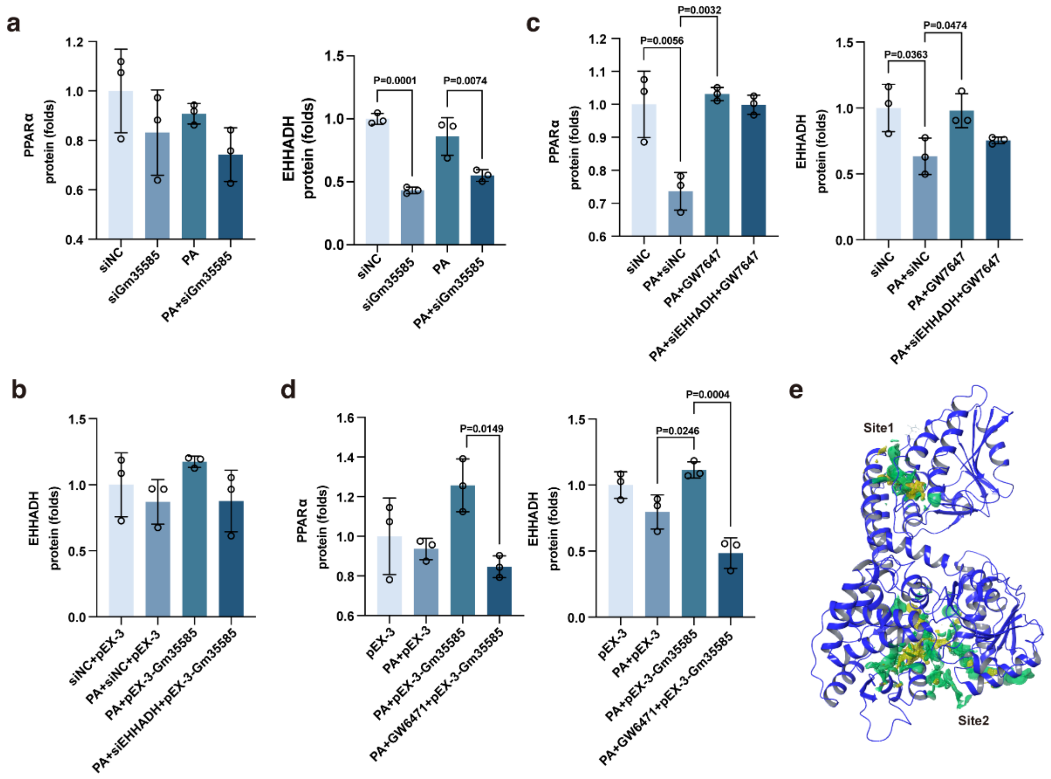

Supplementary Fig. 4. Protein expression levels of PPARα and EHHADH *in vitro* assay.

a Protein expression levels of PPARα and EHHADH under PA, siGm35585 administration. Values were normalized to control group. (mean ± SD, *n* = 3, One-way ANOVA analysis). **b** Protein expression levels of EHHADH under PA, siEHHADH, pEX-3-Gm35585 administration. Values were normalized to control group. (mean ± SD, *n* = 3, One-way ANOVA analysis). **c** Protein expression of PPARα and EHHADH under PA, siEHHADH and GW7647 condition. Values were normalized to control group. (mean ± SD, *n* = 3, One-way ANOVA analysis). **d** Protein expression of PPARα and EHHADH under PA, GW6471 and pEX-3-Gm35585 administration. Values were normalized to control group. (mean ± SD, *n* = 3, One-way ANOVA analysis). **e** Virtual molecular docking of EHHADH crystal structure.

Supplementary Fig. 5

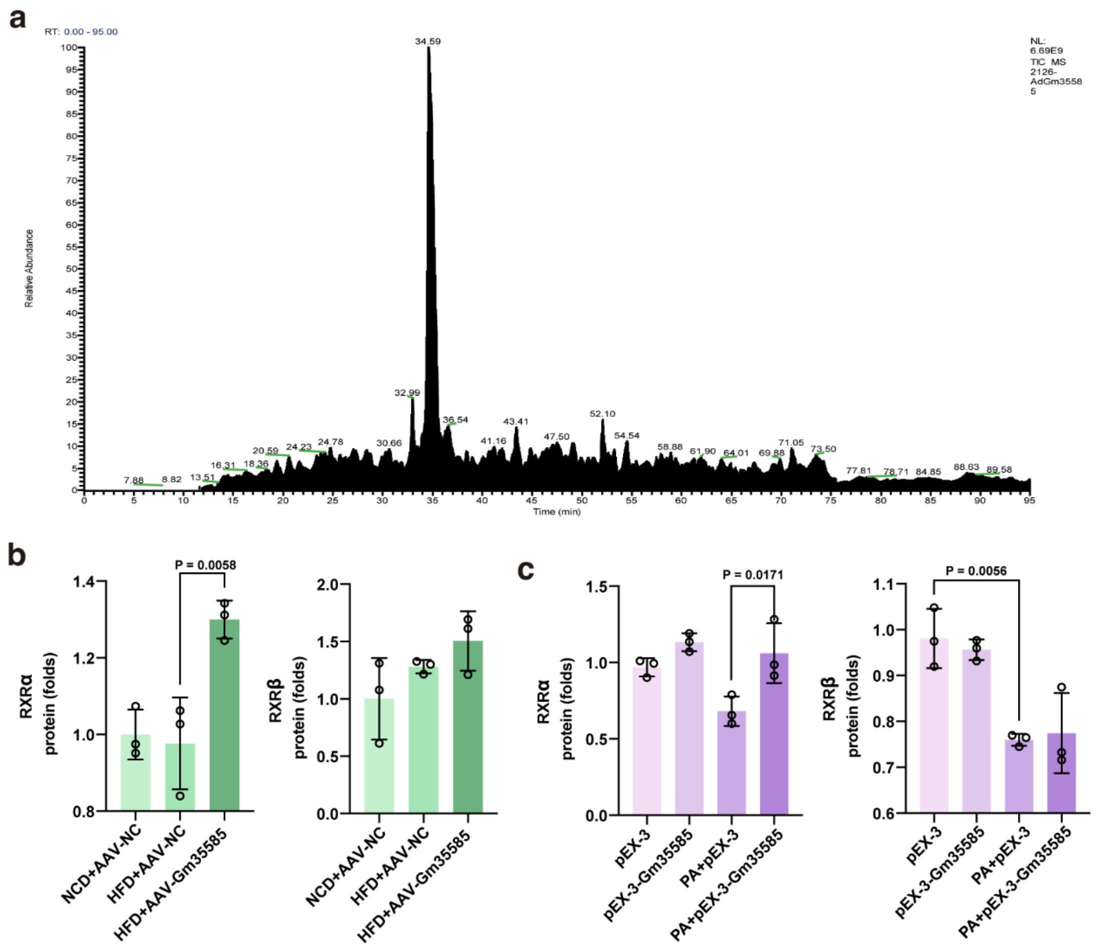

Supplementary Fig. 5. Protein spectrum ion peak diagram of RNA pulldown and protein expression of RXRα and RXRβ.

a Protein spectrum ion peak diagram of RNA pulldown. **b** Protein expression of RXRα and RXRβ *in vivo*. Values were normalized to control group. (mean ± SD, $n = 3$, One-way ANOVA analysis). **c** Protein expression of RXRα and RXRβ *in vitro*. Values were normalized to control group. (mean ± SD, $n = 3$, One-way ANOVA analysis).

Supplementary Fig. 6

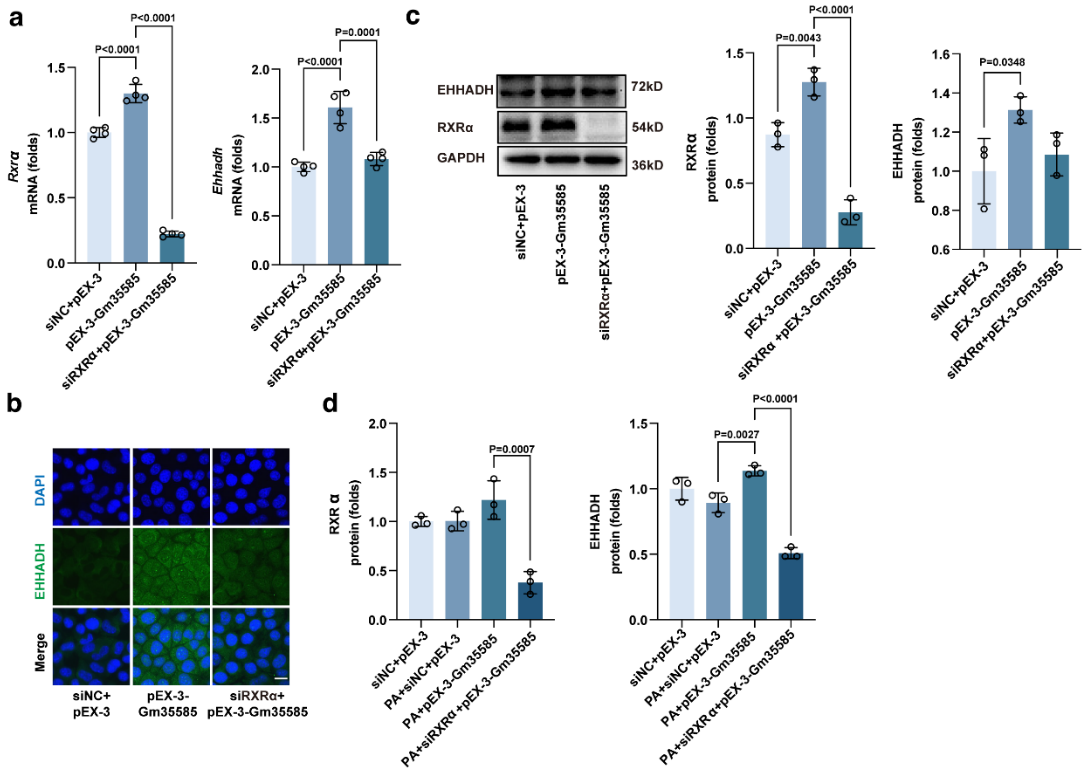

Supplementary Fig. 6. The effect of RXRα silence on EHHADH.

a Under siRXRα and Gm35585 overexpression condition, mRNA expression of *Rxrα* and *Ehhadh*. Values were normalized to control group. (mean ± SD, *n* = 4, One-way ANOVA analysis). **b** EHHADH immunofluorescence staining ahter siRXRα and pEX-3-Gm35585 administration. Scale bar, 25 μm. **c** Protein expression of RXRα and EHHADH. Values were normalized to control group. (mean ± SD, *n* = 3, One-way ANOVA analysis). **d** Protein expression of RXRα and EHHADH *in vitro*. Values were normalized to control group. (mean ± SD, *n* = 3, One-way ANOVA analysis).
